# American Medical Association

**Published:** 1891-12

**Authors:** 


Eugene S. Talbot.

### Domestic Correspondence.

### AMERICAN MEDICAL ASSOCIATION.

[The following circular-note, addressed to the International Dental Journal, seemed to require explanation, and we, therefore, sent it to Dr. E. S. Talbot, ex-president of the Section of Dental and Oral Surgery. His letter in reply accompanied it. In a note subsequently received he states that Dr. S. N. Davis, of Chicago, ex-president of the American Medical Association, and "father of the movement," said, "the letter covered the ground, and that he could not add anything to it."—Ed.]

Philadelphia, August 14, 1891.

Membership in the American Medical Association.—This is obtainable, at any time, by a member of any State or local medical society which is entitled to send delegates to the Association. All that is necessary is for the applicant to write to the treasurer of the Association, Dr. Richard J. Dunglison,

Lock Box 1274, Philadelphia, Pa., sending him a certificate or statement that he is in good standing in his own society, signed by the president and secretary of said society, with five dollars for annual dues. Attendance as a delegate at an annual meeting of the Association is not necessary in order to obtain membership. On receipt of the above amount the weekly journal of the Association will be forwarded regularly.

RICHARD J. DUNGLISON, M.D.,
*Treasurer.*

TO THE EDITOR:

DEAR SIR,—Your letter, asking my opinion in regard to membership in the American Medical Association, is at hand. In reply would say that there has never been a clear understanding among dentists in regard to the resolution adopted by the Association in June, 1887. By this resolution (see August number of INTERNATIONAL DENTAL JOURNAL, page 517) a person who has graduated from a dental college is recognized as a medical practitioner, and is entitled to membership in the American Medical Association, provided the school in which he graduated "requires of its students a standard of preliminary or general education and a term of professional study," etc. (See resolution.) The person must belong to a medical or dental society which has adopted the Code of Ethics of the American Medical Association. Having complied with these requirements, the D.D.S. is eligible to membership on an equal footing with the M.D. The question now is, What colleges have fulfilled these requirements? Although it is not so stated, it was intended that this resolution should include the graduates of such colleges or schools as Harvard, University of Pennsylvania, Ann Arbor, University of Chicago, and other schools that require of their students attendance upon lectures in medical colleges jointly with medical students, and pass the same examination as medical students in the fundamental sciences of medicine, such as anatomy, physiology, chemistry, pathology, and general surgery, omitting such studies as obstetrics, practical medicine, etc., and taking in their place didactic and clinical teaching in dental surgery. So far an exceedingly liberal construction has been put upon this resolution, but if the resolution remains in force in the Association, it will probably not be long before a line will be drawn so that the graduate of no college except those demanding a thorough knowledge of the fundamental principles of a medical education will be admitted to membership. All that is required, then, is to join a medical society which has adopted the Code of Ethics of the American Medical Association, or have some dental society or club adopt the code of ethics, and a person becomes eligible to membership according to the circular of Dr. Dunglison.

I cannot understand, when the best medical men in this country throw open the doors of the National Association to the better class of dentists, why they do not avail themselves of the opportunity and become scientists among scientific men. The medical men have met us more than half-way and are glad to welcome us into their meetings.

Very truly yours,

Eugene S. Talbot.

Chicago, September 28, 1891.

